# 1,2-Bis(4-nitro­benzo­yl)hydrazine

**DOI:** 10.1107/S160053680903181X

**Published:** 2009-08-19

**Authors:** Xue-Yue Jiang, Xiao-Jun Feng, Song Yang, Hua-Jie Xu, Ling-Yun Hao

**Affiliations:** a School of Chemistry & Chemical Engineering, Fuyang Normal College, Fuyang 236041, Anhui, People’s Republic of China; b Department of Biology, Qingyuan Polytechnic, Qingyuan 511515, People’s Republic of China

## Abstract

The title mol­ecule, C_14_H_10_N_4_O_6_, crystallizes with one half-mol­ecule in the asymmetric unit; the mid-point of the N—N bond lies on an inversion centre. The nitro and amide groups are twisted with respect to the benzene ring, making dihedral angles of 14.6 (5) and 31.1 (5)°, respectively. In the crystal structure, mol­ecules are linked through N—H⋯O hydrogen bonding between the imino and carbonyl groups.

## Related literature

For the biological activity of hydrazides, see: Cui *et al.* (2007 ▶); Li & Ban (2009 ▶). For related structures, see: Shang *et al.* (2005*a* ▶,*b* ▶); Zhang *et al.* (2009 ▶).
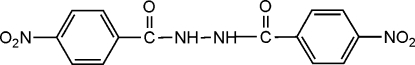


## Experimental

### 

#### Crystal data

C_14_H_10_N_4_O_6_
*M* *_r_* = 330.26
Monoclinic, 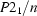

*a* = 4.7947 (6) Å
*b* = 9.8750 (11) Å
*c* = 14.9094 (17) Å
β = 99.05 (3)°
*V* = 697.13 (14) Å^3^
*Z* = 2
Mo *K*α radiation
μ = 0.13 mm^−1^
*T* = 293 K
0.20 × 0.10 × 0.10 mm

#### Data collection

Enraf–Nonius CAD-4 diffractometer
Absorption correction: ψ scan (North *et al.*, 1968 ▶) *T*_min_ = 0.975, *T*_max_ = 0.988
1364 measured reflections
1364 independent reflections
673 reflections with *I* > 2σ(*I*)
3 standard reflections every 200 reflections intensity decay: none

#### Refinement

*R*[*F* ^2^ > 2σ(*F* ^2^)] = 0.070
*wR*(*F* ^2^) = 0.220
*S* = 1.10
1364 reflections
109 parameters
H-atom parameters constrained
Δρ_max_ = 0.13 e Å^−3^
Δρ_min_ = −0.15 e Å^−3^

### 

Data collection: *CAD-4 Software* (Enraf–Nonius, 1989 ▶); cell refinement: *CAD-4 Software*; data reduction: *XCAD4* (Harms & Wocadlo, 1995 ▶); program(s) used to solve structure: *SHELXTL* (Sheldrick, 2008 ▶); program(s) used to refine structure: *SHELXTL*; molecular graphics: *SHELXTL*; software used to prepare material for publication: *SHELXTL*.

## Supplementary Material

Crystal structure: contains datablocks global, I. DOI: 10.1107/S160053680903181X/xu2587sup1.cif

Structure factors: contains datablocks I. DOI: 10.1107/S160053680903181X/xu2587Isup2.hkl

Additional supplementary materials:  crystallographic information; 3D view; checkCIF report

## Figures and Tables

**Table 1:** Hydrogen-bond geometry (Å, °)

*D*—H⋯*A*	*D*—H	H⋯*A*	*D*⋯*A*	*D*—H⋯*A*
N1—H1*A*⋯O1^i^	0.86	2.12	2.881 (5)	147

Symmetry code: (i) 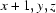.

**Fig. 1.**
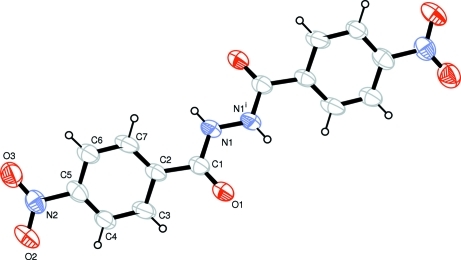
The molecular structure of the title copound, showing 30% probability displacement ellipsoids [symmetry code: (i) 2-x, -y, 1-z].

## supplementary crystallographic information

### Comment

Hydrazides have been demonstrated to possess excellent biological activities (Cui *et al.*, 2007; Li & Ban, 2009). Recently a great deal of hydrazides have been synthesized and characterized (Shang *et al.*, 2005*a*,b; Zhang *et al.*, 2009; Li & Ban, 2009). We also are interested in this field of research, we report here the crystal structure of the title compound.

The molecular structure of the title compound has crystallographically imposed inversion symmetry located in the middle of the N—N bond (Fig. 1). One intermolecular hydrogen bond N—H···O is observed in the crystal structure (Table 1).

### Experimental

4-Nitrobenzohydrazide (0.371 g, 2.0 mmol) and 20 ml chloroform were introduced into a round-bottomed flask at 281 K and stirred. 4-Nitrobenzoyl chloride (0.362 g, 2.0 mmol) was added to the mixture, which was stirred for 2 h at room temperature. A colourless solid product was filtered, and washed three times with ethyl ether. Crystals of the title compound suitable for X-ray structural determination was obtained by slow evaporation a methanol solution in air.

### Refinement

H atoms were placed in geometrically idealized positions and constrained to ride on their parent atoms, with C—H = 0.93 and N—H = 0.86 Å and with *U*_iso_(H) = 1.2 *U*_eq_(C,N).

### Crystal data

**Table:**

C_14_H_10_N_4_O_6_	*F*(000) = 340
*M**_r_* = 330.26	*D*_x_ = 1.573 Mg m^−^^3^
Monoclinic, *P*2_1_/*n*	Mo *K*α radiation, λ = 0.71073 Å
Hall symbol: -P 2yn	Cell parameters from 25 reflections
*a* = 4.7947 (6) Å	θ = 8–12°
*b* = 9.8750 (11) Å	µ = 0.13 mm^−^^1^
*c* = 14.9094 (17) Å	*T* = 293 K
β = 99.05 (3)°	Block, colorless
*V* = 697.13 (14) Å^3^	0.20 × 0.10 × 0.10 mm
*Z* = 2	

### Data collection

**Table:**

Enraf–Nonius CAD-4 diffractometer	673 reflections with *I* > 2σ(*I*)
Radiation source: fine-focus sealed tube	*R*_int_ = 0.0000
graphite	θ_max_ = 26.0°, θ_min_ = 2.5°
ω/2θ scans	*h* = −5→5
Absorption correction: ψ scan (North *et al.*, 1968)	*k* = 0→12
*T*_min_ = 0.975, *T*_max_ = 0.988	*l* = 0→18
1364 measured reflections	3 standard reflections every 200 reflections
1364 independent reflections	intensity decay: none

### Refinement

**Table:**

Refinement on *F*^2^	Primary atom site location: structure-invariant direct methods
Least-squares matrix: full	Secondary atom site location: difference Fourier map
*R*[*F*^2^ > 2σ(*F*^2^)] = 0.070	Hydrogen site location: inferred from neighbouring sites
*wR*(*F*^2^) = 0.220	H-atom parameters constrained
*S* = 1.10	*w* = 1/[σ^2^(*F*_o_^2^) + (0.0632*P*)^2^ + 0.1296*P*] where *P* = (*F*_o_^2^ + 2*F*_c_^2^)/3
1364 reflections	(Δ/σ)_max_ < 0.001
109 parameters	Δρ_max_ = 0.13 e Å^−^^3^
0 restraints	Δρ_min_ = −0.15 e Å^−^^3^

### Special details

**Table:**

Geometry. All e.s.d.'s (except the e.s.d. in the dihedral angle between two l.s. planes) are estimated using the full covariance matrix. The cell e.s.d.'s are taken into account individually in the estimation of e.s.d.'s in distances, angles and torsion angles; correlations between e.s.d.'s in cell parameters are only used when they are defined by crystal symmetry. An approximate (isotropic) treatment of cell e.s.d.'s is used for estimating e.s.d.'s involving l.s. planes.
Refinement. Refinement of *F*^2^ against ALL reflections. The weighted *R*-factor *wR* and goodness of fit *S* are based on *F*^2^, conventional *R*-factors *R* are based on *F*, with *F* set to zero for negative *F*^2^. The threshold expression of *F*^2^ > σ(*F*^2^) is used only for calculating *R*-factors(gt) *etc*. and is not relevant to the choice of reflections for refinement. *R*-factors based on *F*^2^ are statistically about twice as large as those based on *F*, and *R*-factors based on ALL data will be even larger.

### Fractional atomic coordinates and isotropic or equivalent isotropic displacement parameters (Å^2^)

**Table:**

	*x*	*y*	*z*	*U*_iso_*/*U*_eq_	
O1	0.5717 (7)	−0.0146 (4)	0.4016 (2)	0.0940 (11)	
O2	0.8216 (9)	0.1386 (5)	−0.0278 (3)	0.1227 (16)	
O3	1.2216 (11)	0.2315 (5)	0.0270 (3)	0.1219 (15)	
N1	1.0280 (8)	0.0187 (4)	0.4580 (2)	0.0842 (12)	
H1A	1.1951	0.0419	0.4498	0.101*	
N2	1.0017 (12)	0.1715 (5)	0.0358 (3)	0.0949 (13)	
C1	0.8102 (10)	0.0176 (5)	0.3890 (3)	0.0794 (12)	
C2	0.8783 (10)	0.0610 (5)	0.2994 (3)	0.0775 (12)	
C3	0.7163 (12)	0.0022 (6)	0.2224 (4)	0.1007 (16)	
H3A	0.5823	−0.0633	0.2297	0.121*	
C4	0.7531 (11)	0.0399 (6)	0.1371 (3)	0.0920 (15)	
H4A	0.6379	0.0047	0.0865	0.110*	
C5	0.9622 (12)	0.1304 (6)	0.1272 (3)	0.0897 (14)	
C6	1.1218 (12)	0.1918 (5)	0.2030 (4)	0.0926 (15)	
H6A	1.2522	0.2587	0.1952	0.111*	
C7	1.0864 (12)	0.1540 (5)	0.2868 (3)	0.0939 (15)	
H7A	1.2021	0.1903	0.3370	0.113*	

### Atomic displacement parameters (Å^2^)

**Table:**

	*U*^11^	*U*^22^	*U*^33^	*U*^12^	*U*^13^	*U*^23^
O1	0.087 (2)	0.124 (3)	0.0660 (19)	−0.004 (2)	−0.0060 (16)	0.0027 (19)
O2	0.140 (3)	0.145 (4)	0.070 (2)	0.015 (3)	−0.024 (2)	0.009 (3)
O3	0.156 (4)	0.117 (3)	0.090 (3)	−0.013 (3)	0.010 (3)	0.015 (2)
N1	0.077 (2)	0.102 (3)	0.065 (2)	−0.004 (2)	−0.0141 (18)	0.012 (2)
N2	0.117 (3)	0.085 (3)	0.082 (3)	0.014 (3)	0.009 (3)	0.014 (2)
C1	0.086 (3)	0.076 (3)	0.070 (3)	0.000 (2)	−0.007 (2)	0.003 (2)
C2	0.081 (3)	0.079 (3)	0.064 (2)	0.006 (2)	−0.017 (2)	0.005 (2)
C3	0.109 (4)	0.098 (4)	0.080 (3)	−0.017 (3)	−0.029 (3)	0.002 (3)
C4	0.099 (3)	0.102 (4)	0.066 (3)	−0.008 (3)	−0.012 (3)	0.001 (3)
C5	0.113 (4)	0.079 (3)	0.070 (3)	0.017 (3)	−0.009 (3)	0.011 (3)
C6	0.114 (4)	0.075 (3)	0.079 (3)	−0.008 (3)	−0.017 (3)	0.005 (3)
C7	0.112 (4)	0.079 (3)	0.075 (3)	−0.005 (3)	−0.035 (3)	0.003 (3)

### Geometric parameters (Å, °)

**Table:**

O1—C1	1.229 (5)	C2—C3	1.407 (7)
O2—N2	1.221 (6)	C3—C4	1.362 (7)
O3—N2	1.234 (5)	C3—H3A	0.9300
N1—C1	1.346 (6)	C4—C5	1.368 (7)
N1—N1^i^	1.372 (7)	C4—H4A	0.9300
N1—H1A	0.8600	C5—C6	1.400 (7)
N2—C5	1.462 (6)	C6—C7	1.340 (7)
C1—C2	1.488 (6)	C6—H6A	0.9300
C2—C7	1.390 (7)	C7—H7A	0.9300
			
C1—N1—N1^i^	117.1 (5)	C2—C3—H3A	119.6
C1—N1—H1A	121.5	C3—C4—C5	119.0 (5)
N1^i^—N1—H1A	121.5	C3—C4—H4A	120.5
O2—N2—O3	123.8 (5)	C5—C4—H4A	120.5
O2—N2—C5	118.0 (5)	C4—C5—C6	120.8 (5)
O3—N2—C5	118.1 (5)	C4—C5—N2	119.1 (5)
O1—C1—N1	121.0 (4)	C6—C5—N2	119.8 (5)
O1—C1—C2	123.5 (4)	C7—C6—C5	119.9 (5)
N1—C1—C2	115.5 (4)	C7—C6—H6A	120.0
C7—C2—C3	118.6 (5)	C5—C6—H6A	120.0
C7—C2—C1	125.1 (5)	C6—C7—C2	120.6 (5)
C3—C2—C1	116.3 (5)	C6—C7—H7A	119.7
C4—C3—C2	120.9 (5)	C2—C7—H7A	119.7
C4—C3—H3A	119.6		
			
N1^i^—N1—C1—O1	0.7 (8)	C3—C4—C5—N2	−179.8 (5)
N1^i^—N1—C1—C2	179.0 (5)	O2—N2—C5—C4	10.3 (7)
O1—C1—C2—C7	148.0 (5)	O3—N2—C5—C4	−166.2 (5)
N1—C1—C2—C7	−30.3 (7)	O2—N2—C5—C6	−164.5 (5)
O1—C1—C2—C3	−31.9 (7)	O3—N2—C5—C6	19.0 (7)
N1—C1—C2—C3	149.8 (5)	C4—C5—C6—C7	5.5 (8)
C7—C2—C3—C4	−3.0 (8)	N2—C5—C6—C7	−179.8 (5)
C1—C2—C3—C4	176.9 (5)	C5—C6—C7—C2	−4.6 (8)
C2—C3—C4—C5	3.8 (9)	C3—C2—C7—C6	3.4 (8)
C3—C4—C5—C6	−5.0 (8)	C1—C2—C7—C6	−176.5 (5)

Symmetry codes: (i) −*x*+2, −*y*, −*z*+1.

### Hydrogen-bond geometry (Å, °)

**Table:**

*D*—H···*A*	*D*—H	H···*A*	*D*···*A*	*D*—H···*A*
N1—H1A···O1^ii^	0.86	2.12	2.881 (5)	147

Symmetry codes: (ii) *x*+1, *y*, *z*.

## References

[bb1] Cui, Z.-N., Wang, Z., Li, Y., Zhou, X.-Y., Ling, Y. & Yang, X.-L. (2007). *Chin. J. Org. Chem.* **27**, 1300–1304.

[bb2] Enraf–Nonius. (1989). *CAD-4 Software* Enraf–Nonius, Delft, The Netherlands.

[bb3] Harms, K. & Wocadlo, S. (1995). *XCAD4* University of Marburg, Germany.

[bb4] Li, C.-M. & Ban, H.-Y. (2009). *Acta Cryst.* E**65**, o1466. 10.1107/S1600536809020066 PMC2969308 21582769

[bb5] North, A. C. T., Phillips, D. C. & Mathews, F. S. (1968). *Acta Cryst.* A**24**, 351–359.

[bb6] Shang, J., Wang, Q.-M., Huang, R.-Q., Chen, L., Song, H.-B. & Mao, C.-H. (2005*a*). *Acta Cryst.* E**61**, o1043–o1045.

[bb7] Shang, J., Wang, Q.-M., Song, H.-B., Huang, R.-Q., Chen, L. & Mao, C.-H. (2005*b*). *Acta Cryst.* E**61**, o936–o938.

[bb8] Sheldrick, G. M. (2008). *Acta Cryst.* A**64**, 112–122. 10.1107/S0108767307043930 18156677

[bb9] Zhang, M.-J., Yin, L.-Z., Wang, D.-C., Deng, X.-M. & Liu, J.-B. (2009). *Acta Cryst.* E**65**, o508. 10.1107/S1600536809002165 PMC2968684 21582171

